# Electrospun interconnected Fe-N/C nanofiber networks as efficient electrocatalysts for oxygen reduction reaction in acidic media

**DOI:** 10.1038/srep17396

**Published:** 2015-11-30

**Authors:** Nan Wu, Yingde Wang, Yongpeng Lei, Bing Wang, Cheng Han, Yanzi Gou, Qi Shi, Dong Fang

**Affiliations:** 1 Science and Technology on Advanced Ceramic Fibers and Composites Laboratory, National University of Defense Technology, Changsha 410073, P.R. China; 2 College of Basic Education, National University of Defense Technology, Changsha 410073, P. R. China; 3 College of Materials Science and Engineering, Wuhan Textile University, Wuhan 430074, P.R. China

## Abstract

One-dimensional electrospun nanofibers have emerged as a potential candidate for high-performance oxygen reduction reaction (ORR) catalysts. However, contact resistance among the neighbouring nanofibers hinders the electron transport. Here, we report the preparation of interconnected Fe-N/C nanofiber networks (Fe-N/C NNs) with low electrical resistance *via* electrospinning followed by maturing and pyrolysis. The Fe-N/C NNs show excellent ORR activity with onset and half-wave potential of 55 and 108 mV less than those of Pt/C catalyst in 0.5 M H_2_SO_4_. Intriguingly, the resulting Fe-N/C NNs exhibit 34% higher peak current density and superior durability than generic Fe-N/C ones with similar microstructure and chemical compositions. Additionally, it also displays much better durability and methanol tolerance than Pt/C catalyst. The higher electroactivity is mainly due to the more effective electron transport between the interconnected nanofibers. Thus, our findings provide a novel insight into the design of functional electrospun nanofibers for the application in energy storage and conversion fields.

Non-precious metal catalysts (NPMCs) for oxygen reduction reaction (ORR), which are more abundant, less expensive and more durable than the *state-of-the-art* Pt-based catalysts, have demonstrated significantly high activity and enormous potential in the commercialization of proton-exchange membrane fuel cells (PEMFCs)^1^,^2^,^3^,^4^,^5^. At present, Nafion-based PEMFCs can only be operated in acidic environment, exhibiting more mature industrialization than alkaline fuel cells. Thus, the development of effective electrocatalysts for acidic PEMFCs has practical significance. Particularly, catalysts with transition metal (M = Fe, Ni, Co, Mn, *etc*.) coordinating to heterocyclic nitrogen supported on miscellaneous carbon materials, are considered to be the most promising NPMCs in acidic media^6^. Moreover, recent reports also revealed that metal iron and Fe_3_C nanoparticles encased by the graphitic layers demonstrated outstanding ORR activity and stability^7^. Although the exact nature and precise active sites of transition-metal based NPMCs have not been clearly understood yet, both experimental studies and theoretical calculations show that nitrogen-modified carbon and/or M-N_*x*_ moieties play essential roles in ORR^8^,^9^,^10^. Hence, to develop highly active and stable catalysts, extensive research has mainly focused on the selection of nitrogen precursors to promote the formation of effective nitrogen-containing functional groups. The graphitic carbon nitride (g-C_3_N_4_) is constructed from tri-*s*-triazine units with six nitrogen long-pair electrons and planar amino groups^11^. It has been found widespread applications in the sustainable energy fields such as photochemical splitting of water, CO_2_ reduction and electrocatalysis^12^,^13^,^14^,^15^,^16^. Recently, g-C_3_N_4_ has also been chosen as an ideal nitrogen source to integrate with iron ions or incorporate into carbon substrate through thermal decompositon, forming perfect Fe-N/C active sites for ORR^8^,^17^.

To date, a variety of carbon materials have been investigated and applied as ORR catalysts, including carbon nanotubes, graphene, carbon nanofibers and mesoporous carbon^18^,^19^,^20^,^21^,^22^. Among these materials, perfect interconnection of nanofibers into three-dimensional (3D) conductive networks has been found to be critical to facilitate the transport of electron and electrolyte ions^23^,^24^, resulting in high electrochemical performance of fiber catalysts. For instance, Ye and co-workers have prepared a “vein-leaf” type 3D conductive framework of carbon nanofibers in combination with nitrogen-doped graphene to enhance the charge transport^25^. Wu *et al.* have successfully fabricated iron carbide encapsulated in interlinked Fe-N-doped carbon nanofibers with high electronic conductivity and electroactivity^26^.

Electrospinning is considered as a versatile method to produce 3D continuous nanofibers on a large scale^27^,^28^,^29^. Generally, traditional electrospun nanofibers with interlaced nodes can bring contact resistance between the neighbouring fibers. Kadla and co-workers have found that thermally induced inter-fiber bonding was an effective strategy for increasing electronic conductivity^30^. The interconnected bondings were achieved by selecting precursors with different thermal mobilities. Moreover, according to our previous works, electrospun fibers with radial gradient composition or hierarchically porous structure could be fabricated by tuning the thermal treatment precedure and the environment parameters^31^,^32^. However, constructing seamlessly interconnected 3D electrospun nanofiber networks through a simple and effective way is still a huge challenge.

In this work, we report the synthesis of 3D interconnected Fe-N/C nanofiber networks (Fe-N/C NNs) by a novel method. The nanofiber hybrids demonstrate an interconnected framework with large pore channels, considerable active sites and high specific surface area. On the basis of the electrochemical measurements, we found that Fe-N/C NNs displayed a higher diffusion current density, more positive half-wave potential, better stability and greater electron-transfer number than traditional electrospun Fe-N/C nanofiber mats (Fe-N/C NMs) in acidic media. Moreover, Fe-N/C NNs exhibit comparable activity, better durability and methanol tolerance to commercial Pt/C catalyst.

## Results and Discussion

**Figure 1:**
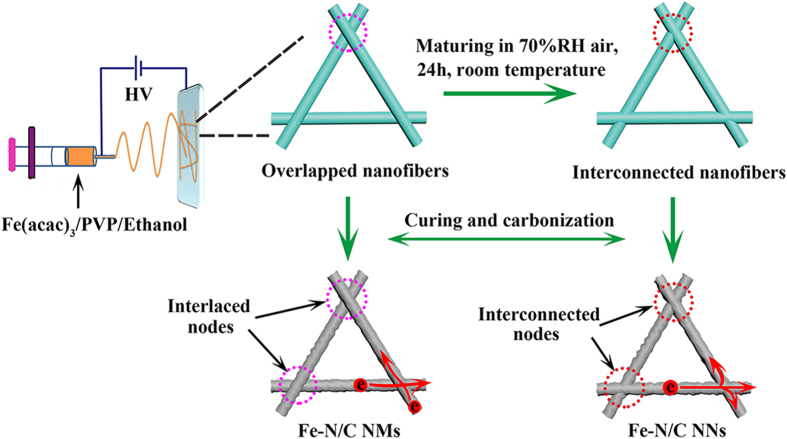
Schematic illustration of the fabrication process for Fe-N/C NNs and Fe-N/C NMs.

The fabrication process of Fe-N/C electrocatalysts is summarized in Fig. 1. The Fe(acac)_3_/PVP nanofibers were produced by electrospinning. After maturing in 70% relative humidity (RH) air for 24 h, Fe-N/C NNs with large amounts of interconnected nodes were synthesized followed by curing and subsequent carbonization. Without maturing process, overlapped Fe-N/C NMs were obtained.

**Figure 2:**
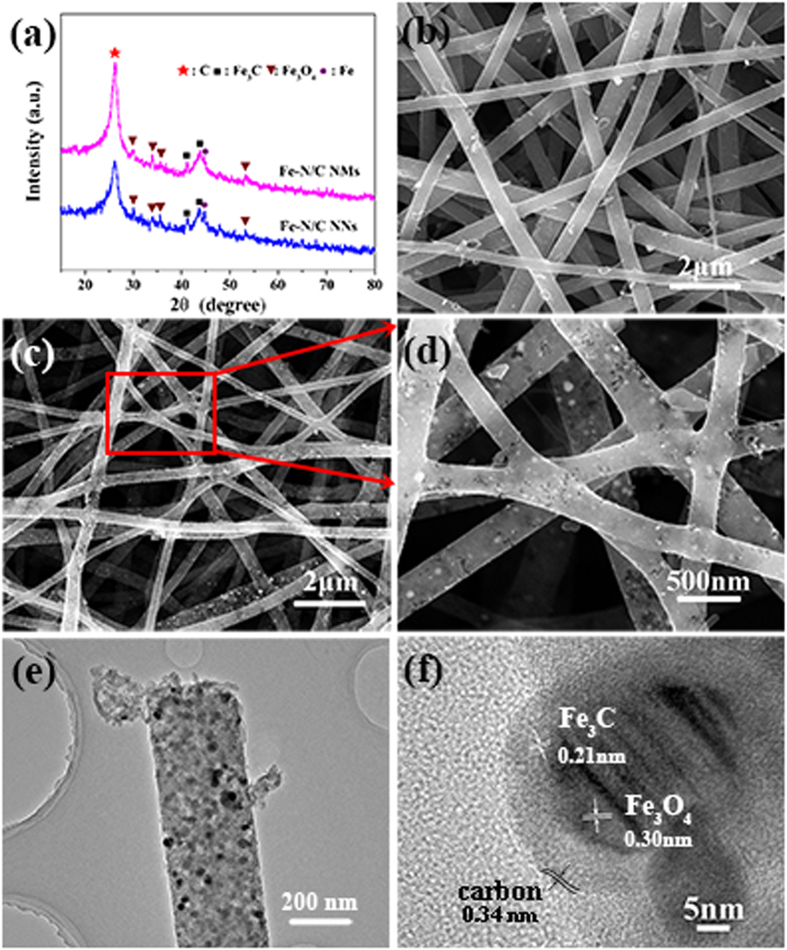
(**a**) XRD patterns of Fe-N/C NNs and Fe-N/C NMs. SEM images of (**b**) Fe-N/C NMs and (**c,d**) Fe-N/C NNs. (**e**) Typical TEM and (**f**) HRTEM images of Fe-N/C NNs revealing the iron compound nanoparticles enchased into the carbon nanofibers.

The structure and morphology of the as-prepared nanofiber samples were investigated by X-ray diffraction (XRD), scanning electron microscopy (SEM) and transmission electron microscopy (TEM). XRD patterns (Fig. 2a) suggest the presence of Fe_3_O_4_ (JCPDS, No. 65–3107), Fe_3_C (JCPDS, No. 65–2412) and α-Fe (JCPDS, No. 65–4899) in the Fe-N/C hybrid nanofibers. Furthermore, the diffraction peak of α-Fe in Fe-N/C NNs becomes weaker remarkably compared with the peak before acid leaching (Fig. S1†), indicating that the exposed unstable α-Fe phase was efficiently removed after being preleached in hot H_2_SO_4_ solution. In addition, the rod-like metal iron crystals on the surface of composite nanofibers (Fig. S2†) disappeared after acid leaching (Fig. 2b-d), which also validated that α-Fe phase was removed. SEM image in Fig. 2b shows that Fe-N/C NMs consist of overlapped, continuous and randomly oriented nanofibers with diameter in the range of 400–500 nm. Interestingly, large numbers of interconnected nodes were obviously observed in the SEM images of Fe-N/C NNs (Fig. 2c,d and Fig. S3†). As analyzed from Fig. S4†, the interconnected system was derived from the exposure of the as-spun Fe(acac)_3_/PVP nanofibers under moist atmosphere. We deduced that the as-spun nanofibers became softening and possessed a high mobility after the absorption of enough water molecules. Then fusion occurred at the intersections while other parts still maintained the fibrous form. TEM image (Fig. 2e) reveals uniform iron-containing nanoparticles embedded into the nanofibers, which could suppress the agglomeration of nanoparticles. Closer inspection by high-resolution TEM (HRTEM, Fig. 2f) displays that Fe_3_O_4_ and Fe_3_C nanoparticles are surrounded by carbon shell. This core-shell structure will protect the iron-based composition from dissolving in acid. The formation of Fe_3_C can be attributed to carbothermal reduction of carbon with iron oxide^33^.

**Figure 3:**
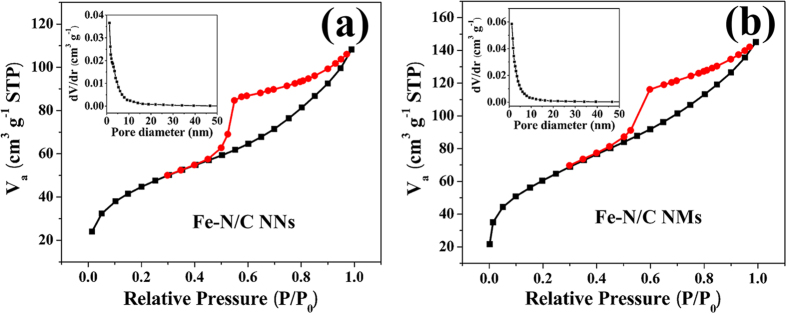
N_2_ sorption isotherms and corresponding pore size distribution curves (inset) for (a) Fe-N/C NNs and (b) Fe-N/C NMs.

Fe-N/C catalysts were further investigated by N_2_ adsorption-desorption techniques. Brunauer-Emmett-Teller (BET) surface areas for Fe-N/C NNs and Fe-N/C NMs are 159.9 and 217.1 m^2^ g^−1^, respectively (Fig. 3). Besides, the remarkable hysteresis loops at the relative pressure range from 0.5 to 1.0 present the mesoporous nature existing in these two samples, which is favourable to the adsorption and transportation of oxygen^34^,^35^. Furthermore, the electrochemical double-layer capacitance (C_dl_), which is considered to be positively proportional to electrochemical active surface area, is determined by applying cyclic voltammograms (CVs) at a series of scan rate^36^. As shown in Fig. S5†, the C_dl_ of Fe-N/C NNs is 9.7 mF cm^−2^, which is larger than that of Fe-N/C NMs (8.2 mF cm^−2^). The higher C_dl_ will provide more effective active sites to enhance the eletrocatalytic performance for Fe-N/C NNs. In addition, the electrical conductivity of Fe-N/C NNs and Fe-N/C NMs measured by two-point probe method (Fig. S6†) is 20.2 and 8.5 S cm^−1^, respectively. This displays that the interconnected framework could provide multidimensional pathways to facilitate electron transport.

**Figure 4:**
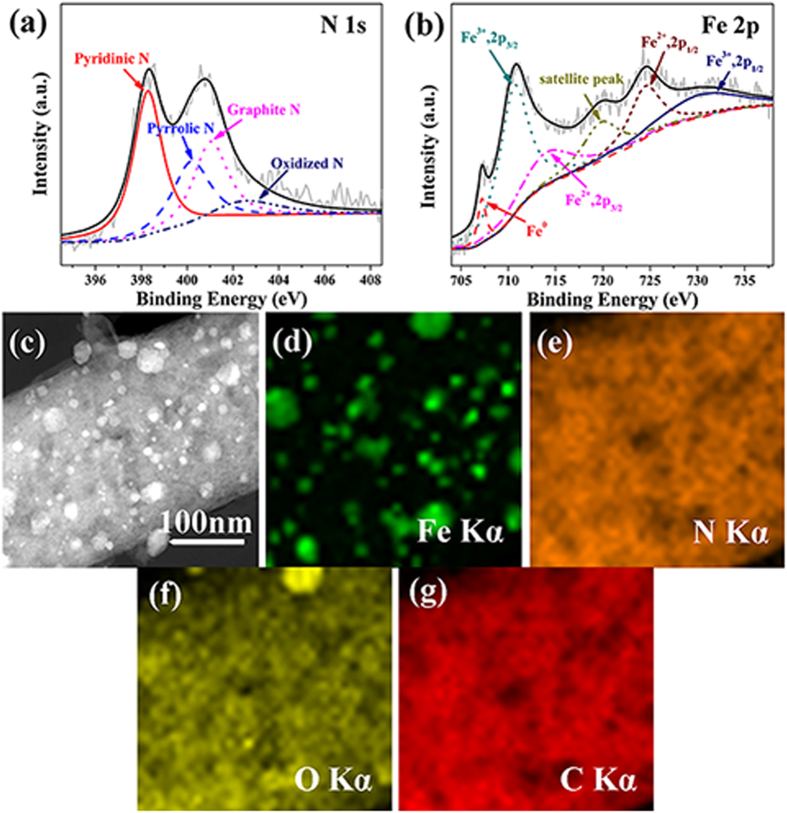
XPS, TEM and elemental analysis of Fe-N/C NNs. High-resolution XPS spectra of (**a**) N 1s and (**b**) Fe 2p. (**c**) Scanning TEM image and corresponding elemental mapping images of (**d**) Fe, (**e**) N, (**f**) O and (**g**) C.

X-ray photoelectron spectroscopy (XPS) analysis was performed to investigate the content and chemical state of nitrogen and iron in the Fe-N/C catalysts. As detected from the survey scans (Fig. S7a and b†), both Fe-N/C NNs and Fe-N/C NMs contain four kinds of elements, carbon, nitrogen, oxygen and iron. In fact, quantum calculations and experimental studies conclude that nitrogen heteroatom may improve the oxygen adsorption and hydrophilicity of the catalyst surface, which can attract electrons readily to enhance the ORR performance^37^,^38^. Moreover, previous reports also revealed that both pyridinic N coordinated with iron and graphitic N contributed mostly to the increase of the ORR performance^39^,^40^. The high-resolution N 1s spectrum for Fe-N/C NNs in Fig. 4a is divided into four species at 398.3, 399.7, 400.8 and 402.0 eV, which can be assigned to pyridinic N (31.4%), pyrrolic N (18.0%), graphitic N (36.5%) and pyridinic oxide N (14.1%), respectively. Both samples show high percentage of total nitrogen content and two kinds of active nitrogen groups (Fig. S7c† and Table. S1†). More effective nitrogen species will donate more active sites to boost the catalytic property. Additionally, Xu^6^ and Sun^8^ *et al.* have reported that Fe (III) and Fe (II) species as active phase play a major role in the superior ORR activity of Fe-N/C catalysts. It can be clearly noted in Fe 2p spectra (Fig. 4b and Fig. S7d†) that Fe (III) and Fe (II) species co-exist in both as-prepared catalysts. Then, scanning TEM and elemental mapping were acquired to further analyze the distribution of species in Fe-N/C NNs (Fig. 4c–g). Interestingly, the O element signal becomes stronger (Fig. 4f) in the region of intensive distribution of Fe element (Fig. 4d), which is accordant with the presence of Fe_3_O_4_ nanoparticles. The homogeneously dispersive N species (Fig. 4e) can bond with the neighbouring C or Fe atoms to provide numerous available active centres for ORR. As can be seen from the Raman spectra (Fig. S8†), the Fe-N/C catalysts display a similar I_*D*_/I_*G*_ value (0.98 for Fe-N/C NNs *vs*. 0.97 for Fe-N/C NMs), indicating analogous level of defect sites in the obtained carbon^41^,^42^.

**Figure 5:**
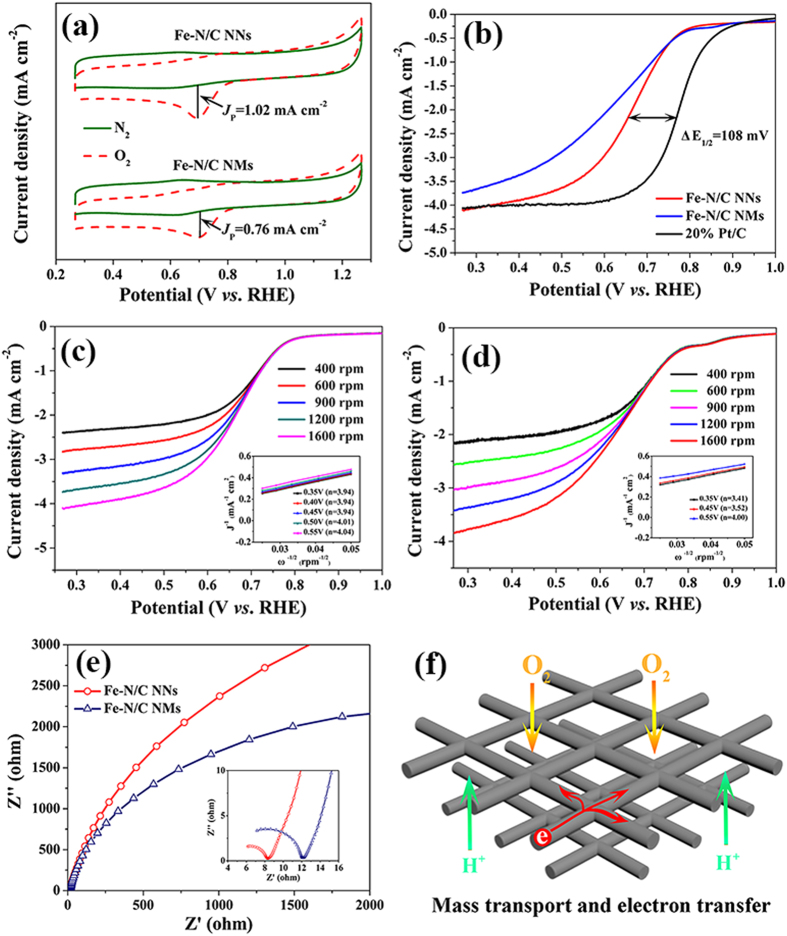
(**a**) CVs of Fe-N/C NNs and Fe-N/C NMs in O_2_- and N_2_-saturated 0.5 M H_2_SO_4_ solution. (**b**) ORR polarization curves of different Fe-N/C catalysts and 20% Pt/C in O_2_-saturated 0.5 M H_2_SO_4_ at 1600 rpm. ORR polarization curves of (**c**) Fe-N/C NNs and (**d**) Fe-N/C NMs in O_2_-saturated 0.5 M H_2_SO_4_ at different rotation rates. Inset is the corresponding K-L plots at a potential range from 0.35 to 0.55 V. (**e**) Nyquist plots of Fe-N/C NNs and Fe-N/C NMs. (**f**) Scheme of interconnected Fe-N/C NNs facilitating mass and electron transport.

To evaluate the electrochemical activity of Fe-N/C NNs and Fe-N/C NMs, a series of CVs were carried out in N_2_- and O_2_-saturated 0.5 M H_2_SO_4_ solution at a scan rate of 10 mV s^−1^ (Fig. 5a). One can see that the two samples exhibit a well-defined cathodic peak at around 0.7 V in O_2_-saturated H_2_SO_4_ solution. To correct the background current, the featureless voltammogram recorded in N_2_-saturated 0.5 M H_2_SO_4_ solution is subtracted from the voltammogram recorded in O_2_-saturated electrolyte. It is well known that a higher peak current density (*J*_P_) is beneficial to a better ORR performance. The *J*_P_ of Fe-N/C NNs (1.02 mA cm^−2^) is significantly 34% higher than that of Fe-N/C NMs (0.76 mA cm^−2^). The higher *J*_P_ of Fe-N/C NNs correlates well with the interconnected nanofiber networks, which provide continuous pathways for electron transport^37^.

To gain further insight into the ORR activity of Fe-N/C catalysts in comparison with commercial Pt/C catalyst, linear sweep voltammograms (LSVs) were recorded on a rotating disk electrode (RDE) in an O_2_-saturated electrolyte. As depicted in Fig. 5b, the onset and half-wave potential (E_1/2_) of Fe-N/C NNs derived from LSVs are 0.858 and 0.662 V, respectively, demonstrating 55 and 108 mV less than those of Pt/C catalyst. Whereas E_1/2_ of Fe-N/C NNs shows 35 mV more positive than that of Fe-N/C NMs. In particular, the diffusion-limiting current density also reveals a better electrochemical value of Fe-N/C NNs (*e.g*., 4.05 mA cm^−2^ at 0.3 V) than that of Fe-N/C NMs (3.66 mA cm^−2^ at 0.3 V). Note that the electroactivity of Fe-N/C NNs is still not as good as Pt/C catalyst in terms of onset potential and E_1/2_. However, Fe-N/C NNs exhibit comparable performance to the recent good results in acidic media (Table. S2†).

LSVs collected at various rotation speeds of different samples were used to determine the electron transfer number (*n*) on the basis of Koutechy-Levich (K-L) plots. The corresponding *n* value for Fe-N/C NNs is calculated to be around 4 over the potential range from 0.35 to 0.55 V (Fig. 5c), representing an efficient four-electron (4e^−^) dominated ORR process similar to Pt/C catalyst. Nevertheless, the *n* value for Fe-N/C NMs varied strongly with the electrode potential (Fig. 5d). Remarkably, a 4e^−^ reduction pathway will decrease the H_2_O_2_ yield, which can lead to a considerable stability. To investigate the electrode kinetics under ORR process for various catalysts, electrochemical impedance spectroscopy (EIS) measurements were performed at their corresponding open circuit voltage. As demonstrated in Nyquist plots (Fig. 5e), Fe-N/C NNs exhibit a smaller interfacial and charge-transfer resistance (*R*_*ct*_, 4.3 Ω) than Fe-N/C NMs (*R*_*ct*_, 7.1 Ω). The lower *R*_*ct*_ of Fe-N/C NNs will markedly facilitate the process for shuttling charges from electrocatalysts to oxygen. Additionally, the net-like structure with continuous large-pores, constructed from entangling of different nanofibers, can facilitate the penetration of electrolyte during the ORR process (Fig. 5f)^43^. Taken together, the superior ORR activity of Fe-N/C NNs could be mainly attributed to the interconnected nanofiber networks which boosted the mass transport and electron transfer.

**Figure 6:**
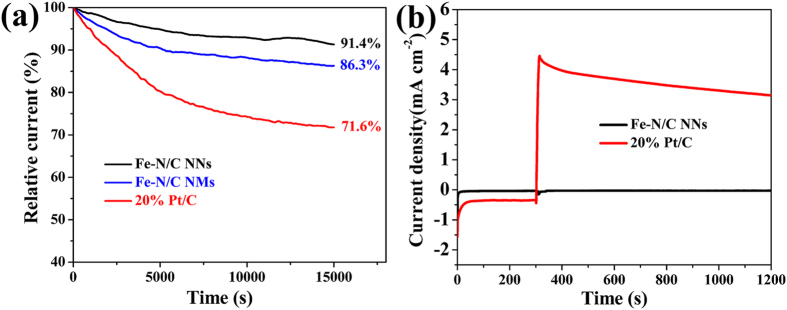
(**a**) Chronoamperometric response of Fe-N/C NNs, Fe-N/C NMs and Pt/C in O_2_-saturated 0.5 M H_2_SO_4_ at 0.75 V. (**b**) Chronoamperometric response with 3 M methanol in O_2_-saturated 0.5 M H_2_SO_4_ at 0.75 V.

Along with the excellent ORR activity, durability and tolerance towards the methanol crossover effect of the catalysts are two important factors for practical applications as well. The current-time (*i-t*) chronoamperometric response in O_2_-saturated electrolyte at 0.75 V (Fig. 6a) indicates that Fe-N/C NNs suffer from a slight attenuation (8.6%) after 15000 s compared with Fe-N/C NMs (13.7%) and Pt/C catalyst (28.4%), suggesting an outstanding durability of Fe-N/C NNs. It is well known that the poor durability of Pt/C catalyst originates from the aggregation of platinum nanoparticles after the oxidation degradation of carbon supports. In our system, the composite structure of iron-containing hybrids embedded in carbon nanofiber will hinder the dissolution and aggregation of active sites, leading to a better stability. As shown in Fig. 6b, the original ORR current of Pt/C catalyst changes dramatically after the addition of 3 M methanol, suggesting the occurrence of the methanol oxidation reaction. In sharp contrast, the chronoamperometric response of Fe-N/C NNs recovers quickly upon the injection of methanol. Indeed, Fe-N/C NNs catalyst is also a promising candidate for the direct methanol fuel cells.

In conclusion, we have successfully developed novel 3D interconnected Fe-N/C nanofiber networks as ORR catalyst *via* a simple maturing process under moist air atmosphere after electrospinning. The interconnected nanofiber structure can provide a continuous and multidimensional pathway to facilitate electron transport. Besides the network structure, the synergistic effect between nitrogen-doped Fe/C complex and high specific area for Fe-N/C NNs play important roles in the excellent ORR activity, superior durability and methanol tolerance. We believe that our method highlights the possibility for the fabrication of other interconnected nanofibers for battery, supercapacitor and fuel cells applications.

## Experimental section

### Materials

All of the chemical reagents were used as received. Polyvinylpyrrolidone (PVP, M_n_=1,300,000) was purchased from Shanghai Dibai Chemical Reagent Co., Ltd. Iron acetylacetonate (Fe(acac)_3_) was supplied by Xiya Reagent Co., Ltd. Methanol (98%), ethanol (99.7%), sulphuric acid (H_2_SO_4_, 98%), isopropyl alcohol and melamine (99.55%) were acquired from Tianjin Guangfu Fine Chemical Reagent Research Institute. Nafion solution (5 wt%, Dupont D520) and Pt/C (20 wt%, JM) were supplied by Shanghai Hesen Electric Co., Ltd. Nitrogen (N_2_) and oxygen (O_2_) with a purity of 99.99% was supplied by Hunan Xianggang Co., Ltd. Deionized (DI) water was produced in our lab.

### Fabrication of Fe-N/C hybrid nanofibers

In a typical procedure, 1.0 g PVP and 0.2 g Fe(acac)_3_ were first dispersed into 10 mL ethanol solvent followed by vigorous stirring for 6 h at room temperature. Then the homogeneous precursor solution was transferred into a 10 mL plastic syringe equipped with a needle of 0.8 mm inner diameter. A syringe pump (Longer Pump LSP02-1B, China) was used to keep a constant flow rate of 15 *μ*L min^−1^. A voltage of 15 kV, generated by a power supply (Dongwen High-Voltage Power DW-P303-1ACF0, China), was applied between the needle and the aluminum foil collector at a distance of 15 cm. The electrospinning process was performed at room temperature and about 40% RH.

To prepare Fe-N/C NNs, the as-spun composite nanofibers were matured in 60 ∼ 70% RH air atmosphere for 24 h at room temperature to make the interconnected nanofibers. Then the matured nanofibers were stabilized at 260 °C for 2 h with a heating rate of 3 °C min^−1^. After that, 1.0 g g-C_3_N_4_ and 0.2 g stabilized nanofibers (covered on the g-C_3_N_4_) were loaded in a ceramic crucible and then heated to 900 °C at a rate of 5 °C min^−1^ for 2 h in a tubular furnace (Zhonghuan SK-G08143, China) under N_2_ atmosphere. Fe-N/C NMs were fabricated without the maturing process. The g-C_3_N_4_ was synthesized previously by the pyrolysis of melamine at 550 °C for 4 h.

The as-obtained hybrid nanofibers were then preleached in 0.5 M H_2_SO_4_ solution at 80 °C for 8 h to remove the unstable and inactive species, followed by washing in DI water and drying. Finally, the hybrid nanofibers were heat-treated again to 900 °C at a rate of 5 °C min^−1^.

### Characterizaition

XRD patterns were collected in the range of 15–80° (2θ) using Siemens D-500 diffractometer (Cu Kα radiation, *λ* = 1.5406 Å) working at 40 kV and 40 mA. Field emission SEM (FESEM, Hitachi S-4800, Japan) was used to study the morphology of the hybrid nanofibers. TEM was conducted on a TecnaiTF200 microscopy operating at 200 kV. Elemental mappings of the sample were obtained through the EDAX detector attached to TEM. The TEM samples were prepared by dropping the suspension of the broken nanofibers on copper grids and then drying under ambient conditions. XPS measurements were recorded with an Thermo Scientific Escalab 250xi instrument equipped with a monochromatic Al Kα source. Raman measurement was performed on Bruker RAM II with a laser wavelength of 532 nm. The specific surface area and the pore size distribution of the samples were estimated from nitrogen adsorption isotherm (BELSORP-mini II, Japan) by means of the BET equation and the Barret-Joyner-Halenda (BJH) model, respectively.

### Electrochemical measurements

For the electrochemical test, the Fe-N/C hybrid nanofibers were finely ground to powder in an agate mortar. Then 8 mg catalyst and 40 *μ*L Nafion aqueous solution were dispersed in 750 *μ*L DI water and 250 *μ*L isopropyl alcohol. A homogeneous catalyst ink was obtained by ultrasonicating the above mixture slightly for 1 h. To prepare the working electrode for electrochemical measurements, 5 *μ*L ink was dropped on a mirror polished glass carbon electrode followed by drying in air.

The electrochemical performance of the samples was measured with an electrochemical working station (CHI 660E, CH instrument, China) and a RDE apparatus (RRDE-3A, ALS, Japan) in a conventional three-electrode system. A platinum wire electrode and a saturated calomel electrode (SCE) were used as counter electrode and reference electrode, respectively. SCE was calibrated to reversible hydrogen electrode (RHE) as described in electronic supplementary information (Fig. S9†). The electrolyte for all the tests was 0.5 M H_2_SO_4_ solution.

Cyclic voltammetry experiments (catalyst loading: 0.57 mg cm^−2^) were carried on the electrochemical working station from 1.27 to 0.27 V at a scan rate of 10 mV s^−1^. Before the test, the electrolyte was saturated with N_2_ or O_2_ for 30 min. RDE measurements (catalyst loading: 0.32 mg cm^−2^) were recorded by LSVs in O_2_ saturated 0.5 M H_2_SO_4_ solution from 1.0 to 0.27 V at a scan rate of 10 mV s^−1^ with different rotation rates. Before recording, the working electrode was cycled for 20 cycles to stabilize the current density. The electron transfer number for ORR at catalyst electrodes was determined by the K-L equation (Equation 1):


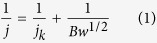


where *j* is the measured current density, *j*_*K*_ is the kinetic current density and *ω* is the electrode rotating rate. The parameter *B* could be calculated from the slope of the K-L plots based on the following Levich equation (Equation 2):


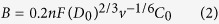


where *n* is the electron transfer number per oxygen molecule, *F* is Faraday constant (*F* = 96485 C mol^−1^), *D*_0_ is the diffusion coefficient of O_2_ in 0.5 M H_2_SO_4_ (*D*_0_ = 1.9 × 10^−5^ cm^2^ s^−1^), *v* is the kinetic viscosity (*v* = 0.01 cm^2^ s^−1^), *C*_0_ is the bulk concentration of O_2_ (*C*_0_ = 1.2 × 10^−6^ mol cm^−3^). The value 0.2 is applied when the rotation speed is expressed in revolutions per minute (rpm).

EIS measurements were performed in O_2_-saturated electrolyte at a frequency range from 100 kHz to 0.01 Hz. The stability performance of the Fe-N/C hybrids was tested at a fixed potential of 0.75 V for the chronoamperometry.

## Additional Information

**How to cite this article**: Wu, N. *et al.* Electrospun interconnected Fe-N/C nanofiber networks as efficient electrocatalysts for oxygen reduction reaction in acidic media. *Sci. Rep.* **5**, 17396; doi: 10.1038/srep17396 (2015).

## Supplementary Material

Supplementary Information
