# Supplementary material for: Electrospun interconnected Fe-N/C nanofiber networks as efficient electrocatalysts for oxygen reduction reaction in acidic media
Source: Sci Rep. 2015 Nov 30;5:17396. doi: 10.1038/srep17396 (PMC4663764; doi:10.1038/srep17396)
Supplement: Supplementary Information [file srep17396-s1.doc]

**Supplementary Information**

**Electrospun interconnected Fe-N/C nanofiber networks as efficient electrocatalystsfor oxygen reduction reaction in acidic media**

Nan Wu^1^, Yingde Wang^1^, Yongpeng Lei^2^, Bing Wang^1^, Cheng Han^1^, Yanzi Gou^1^, Qi Shi^1^ & Dong Fang^3^

*^1^Science and Technology on Advanced Ceramic Fibers and Composites Laboratory, National University of Defense Technology, Changsha 410073, P. R. China*

*^2^College of Basic Education, National University of Defense Technology, Changsha 410073,P. R. China*

*^3^College of Materials Science and Engineering, Wuhan Textile University, Wuhan 430074, P. R. China*

^＊^*Corresponding author:*[*wyd502@163.com*](mailto:wyd502@163.com) *(Y. Wang);* [*lypkd@163.com*](mailto:lypkd@163.com)*(Y. Lei)*

*Fax: 86-731-84575118; Tel: 86-731-84575118*


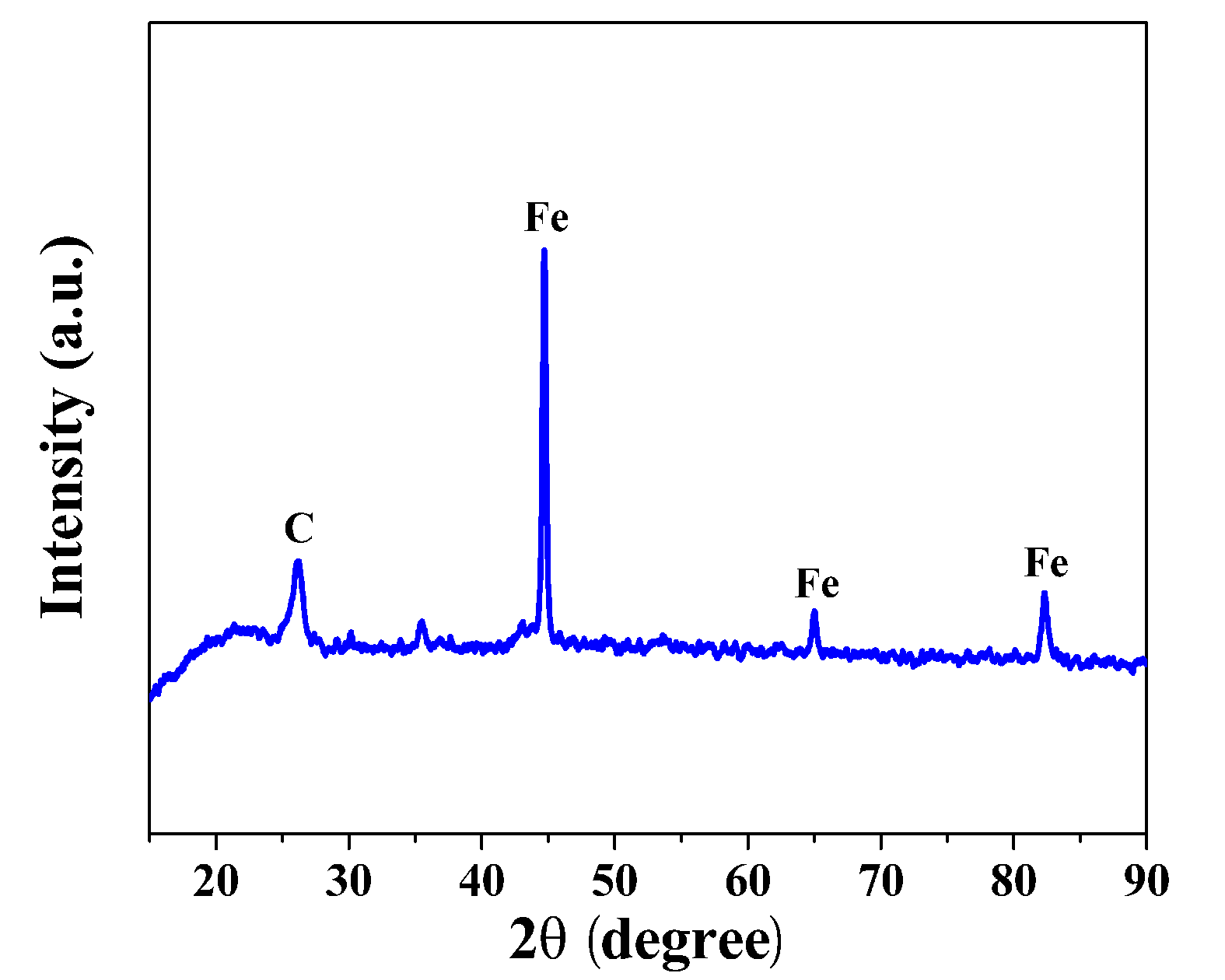


Figure S1. XRD pattern of Fe-N/C NNs before leaching in 0.5M H_2_SO_4_ at 80°C for 8h.


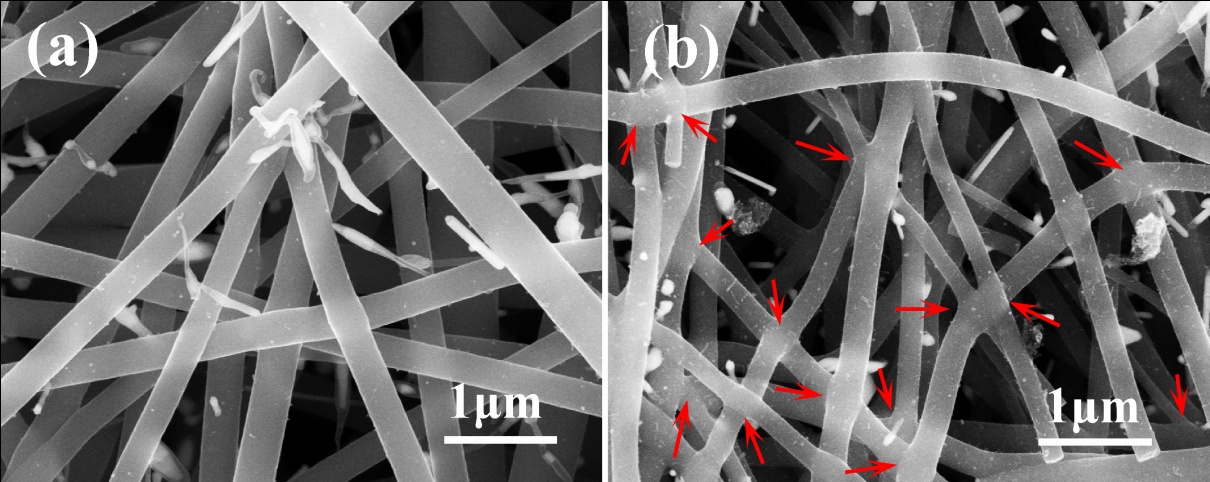


Figure S2. SEM images of (a) Fe-N/C NMs and (b) Fe-N/C NNs before acid leaching. The red arrows in (b) designate the interconnected nodes in Fe-N/C NNs.


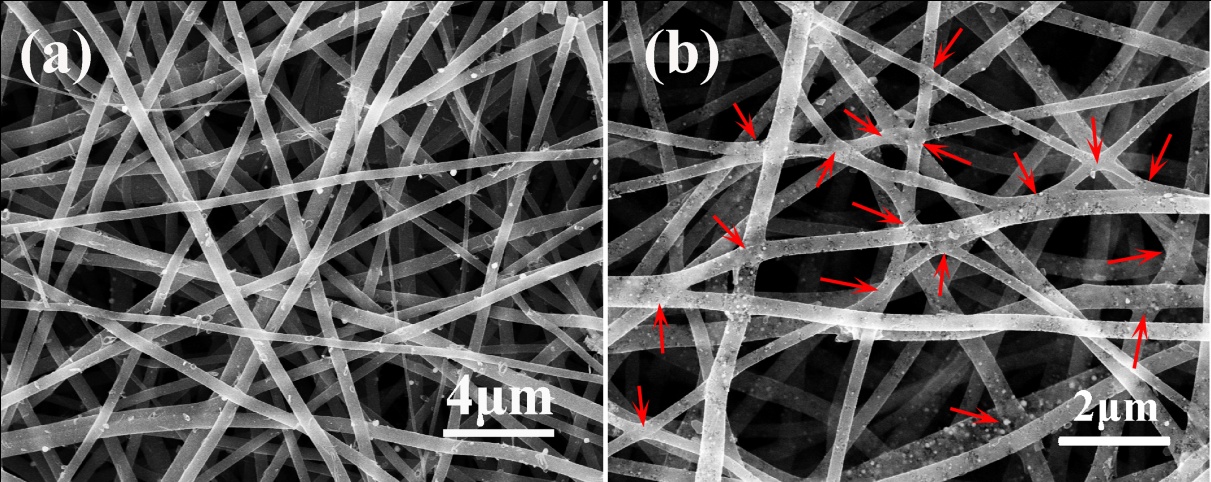


Figure S3. SEM images of (a) Fe-N/C NMs and (b) Fe-N/C NNs after acid leaching. The red arrows in (b) designate the interconnected nodes in Fe-N/C NNs.


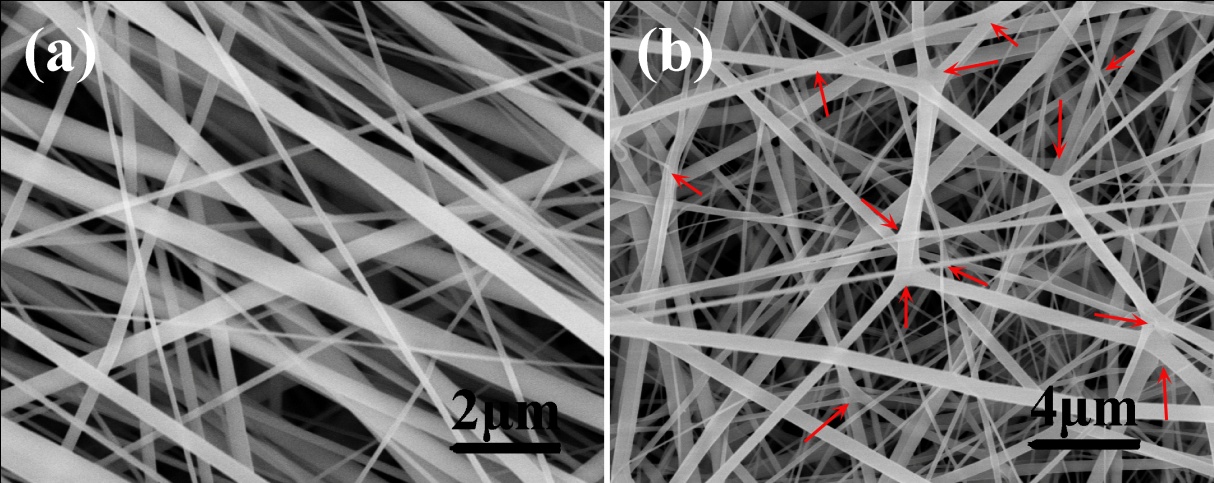


Figure S4. SEM images of (a) as-spun Fe(acac)_3_/PVP and (b) interconnected Fe(acac)_3_/PVP nanofibers after maturing in 70% RH moist atmosphere for 24 h. The red arrows designate the interconnected nodes in interconnected Fe(acac)_3_/PVP nanofibers.


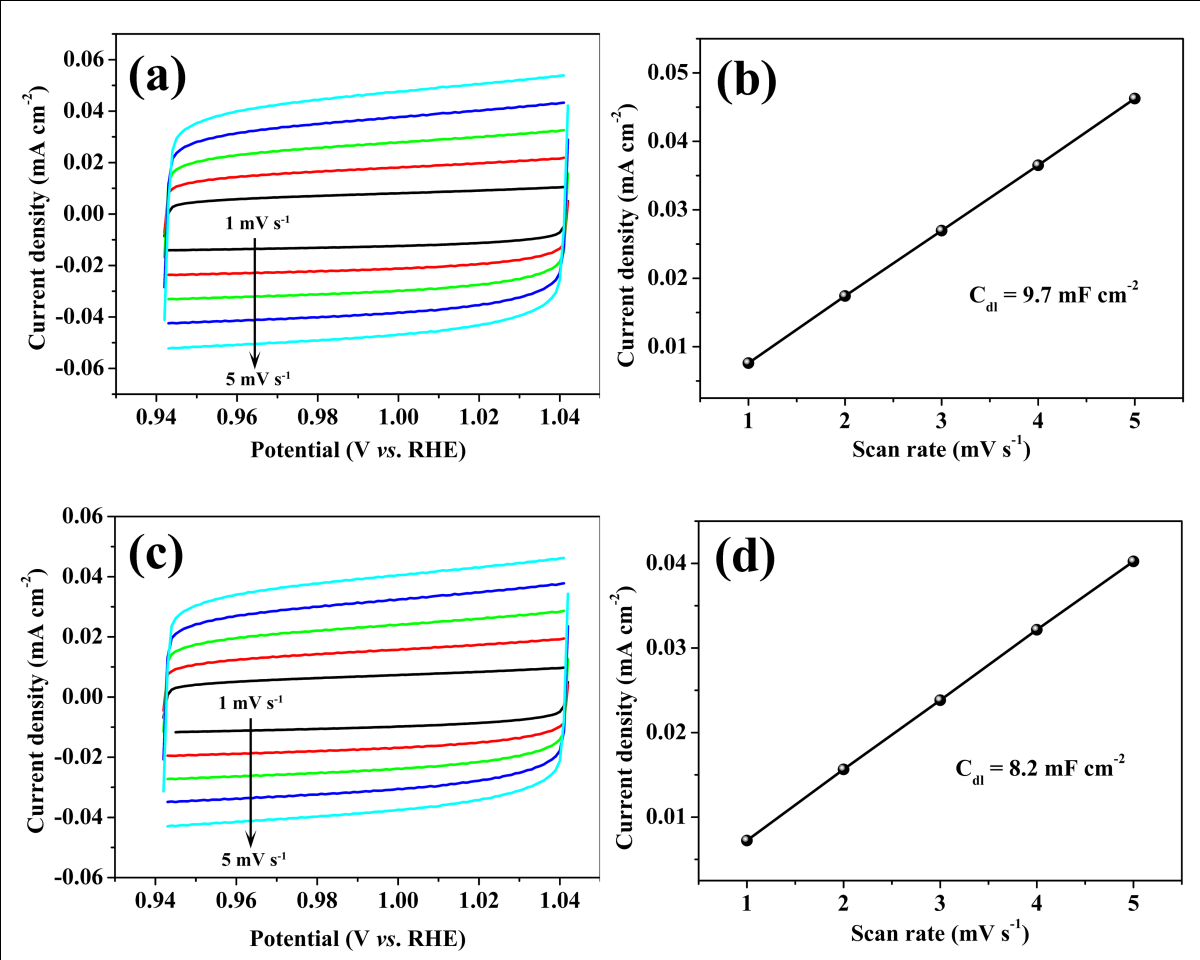


Figure S5. CVs at scan rates of 1, 2, 3, 4 and 5 mV s^-1^ in O_2_-satuated 0.1 M KOH electrolyte and the corresponding current density taken at 0.99 V for (a,b) Fe-N/C NNs and (c,d) Fe-N/C NMs.

The electrochemical double-layer capacitance (C_dl_) was calculated by the following equation:

C_dl_ =Δ*I*_c_/Δ*v*

where C_dl_, *I*_c_ and *v* are the double-layer capacitance (mF cm^-2^), current density (mA cm^-2^) and scan rate (mV s^-1^), respectively.


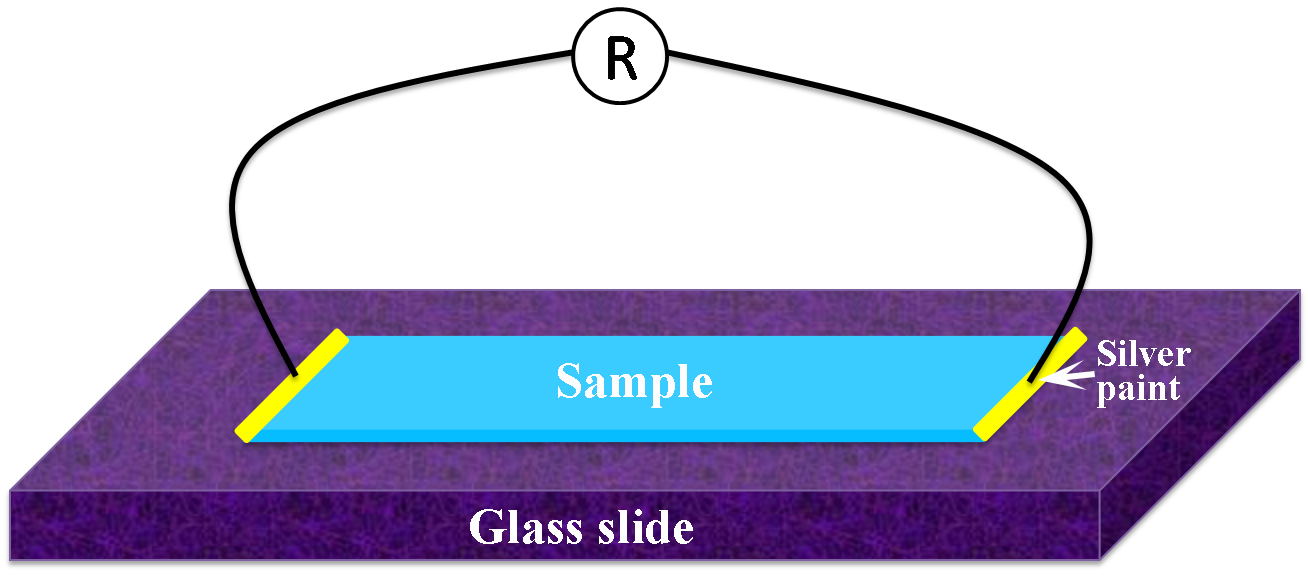


Figure S6. Schematic illustration of electrical conductivity measurement method.

As shown in Fig. S6, the samples were painted at each end with silver paint and mounted onto glass slides. The size of sample was 1.5 cm in length and 0.5 cm in width. Conductivity *κ* (S cm^-1^) was calculated by the following equation：

*κ = l/ (Rtw)*

where *l*, *t* and *w* was the length, thickness and width (cm) of the sample, respectively. The thickness was measured by a micrometer. The resistance *R* (Ω) of samples was measured by a multimeter.


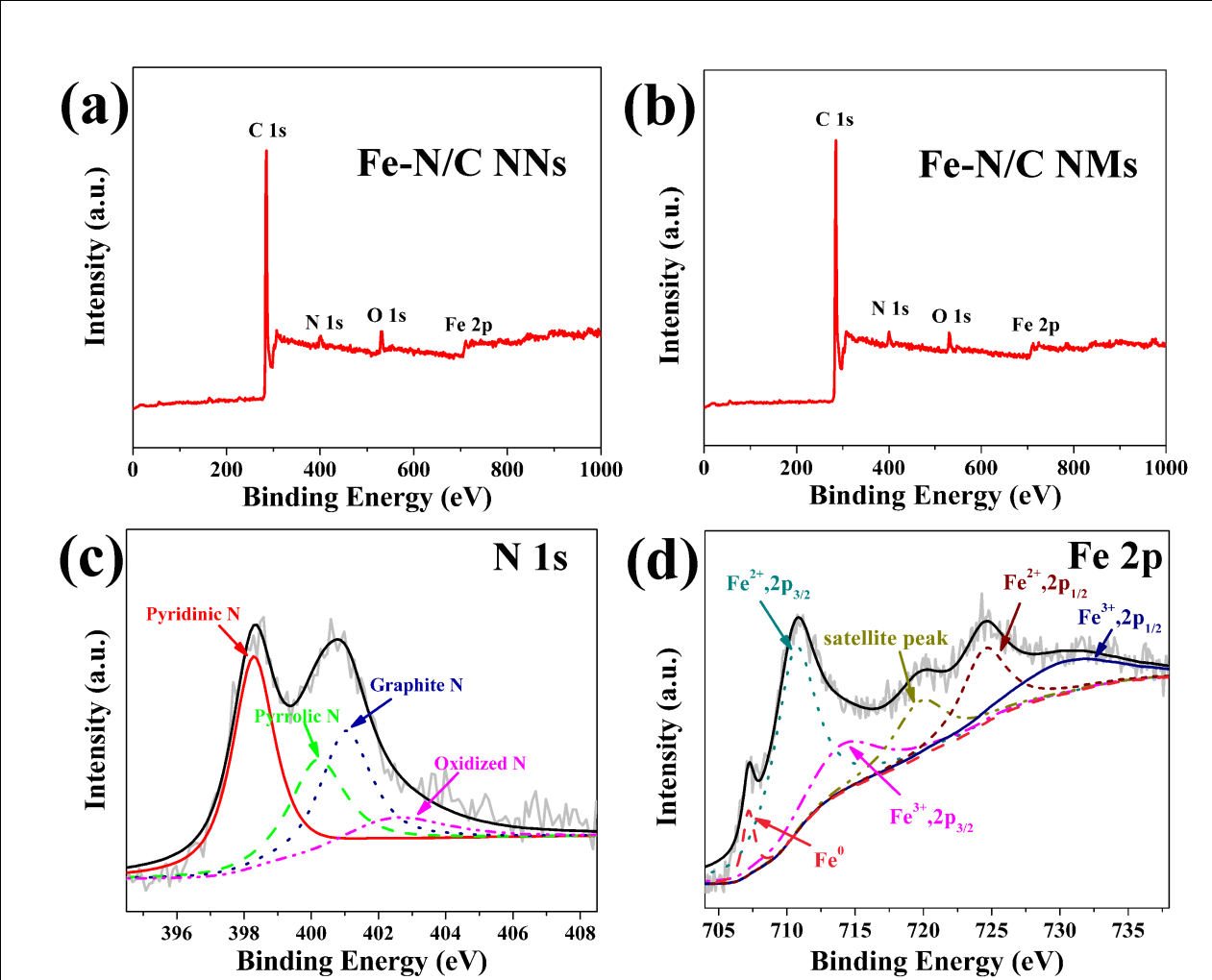


Figure S7. XPS survey spectra of (a) Fe-N/C NNs and (b) Fe-N/C NMs. High resolution spectra of (c) N 1s and (d) Fe 2p for Fe-N/C NMs.


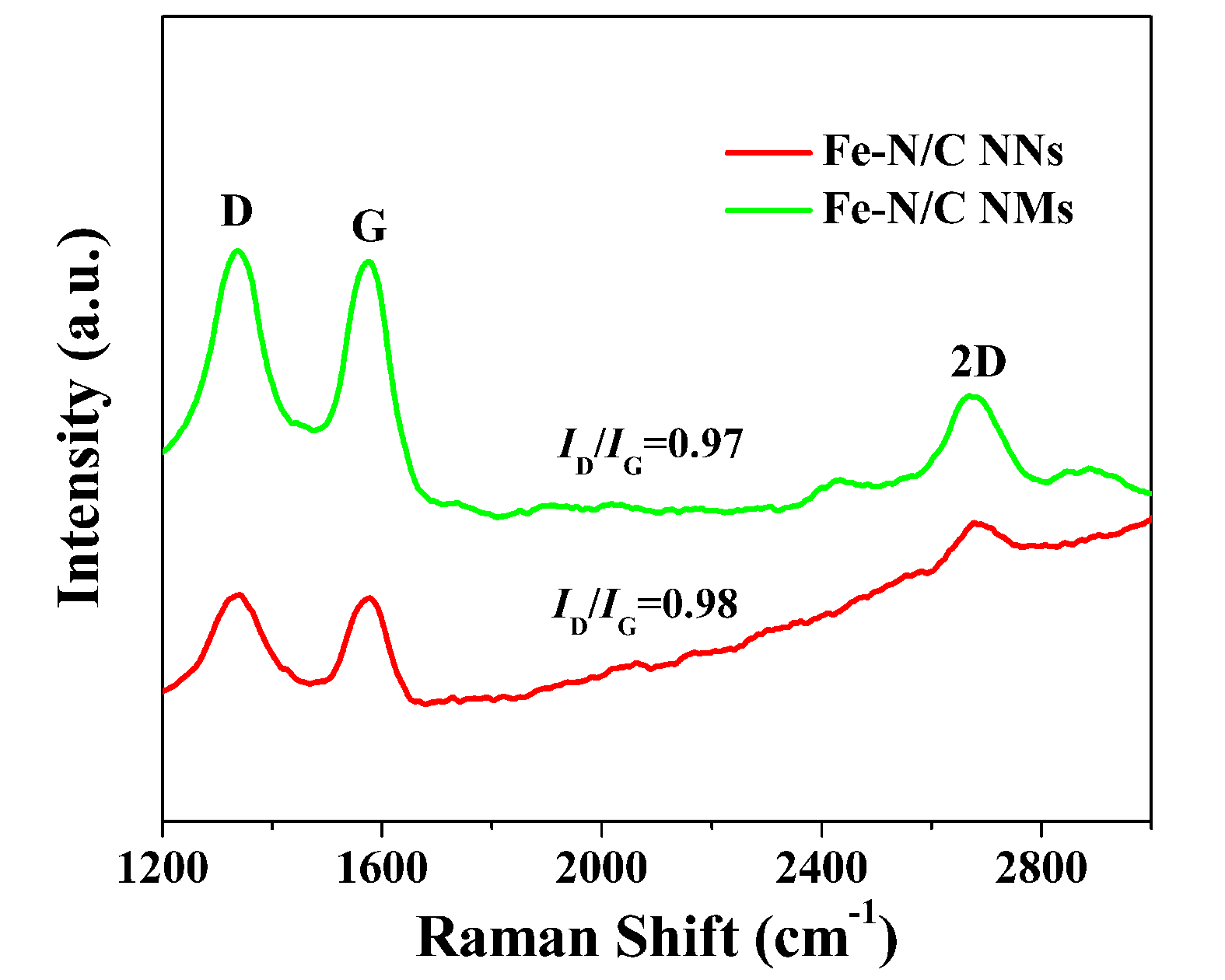


Figure S8. Raman spectra of Fe-N/C NNs and Fe-N/C NMs.

**RHE calibration**

The calibration was performed in the hydrogen saturated electrolyte with 20% Pt/C nanoparticles (Hispec 3000) as the working electrode. The average potential at which the current crossed zero was taken to be the thermodynamic potential for the hydrogen electrode reactions. CV was measured at a scan rate of 1 mV s^-1^ (Figure. S9). Thus, in 0.5 M H_2_SO_4_ solution, E(RHE) = E(SCE) + 0.267 V.


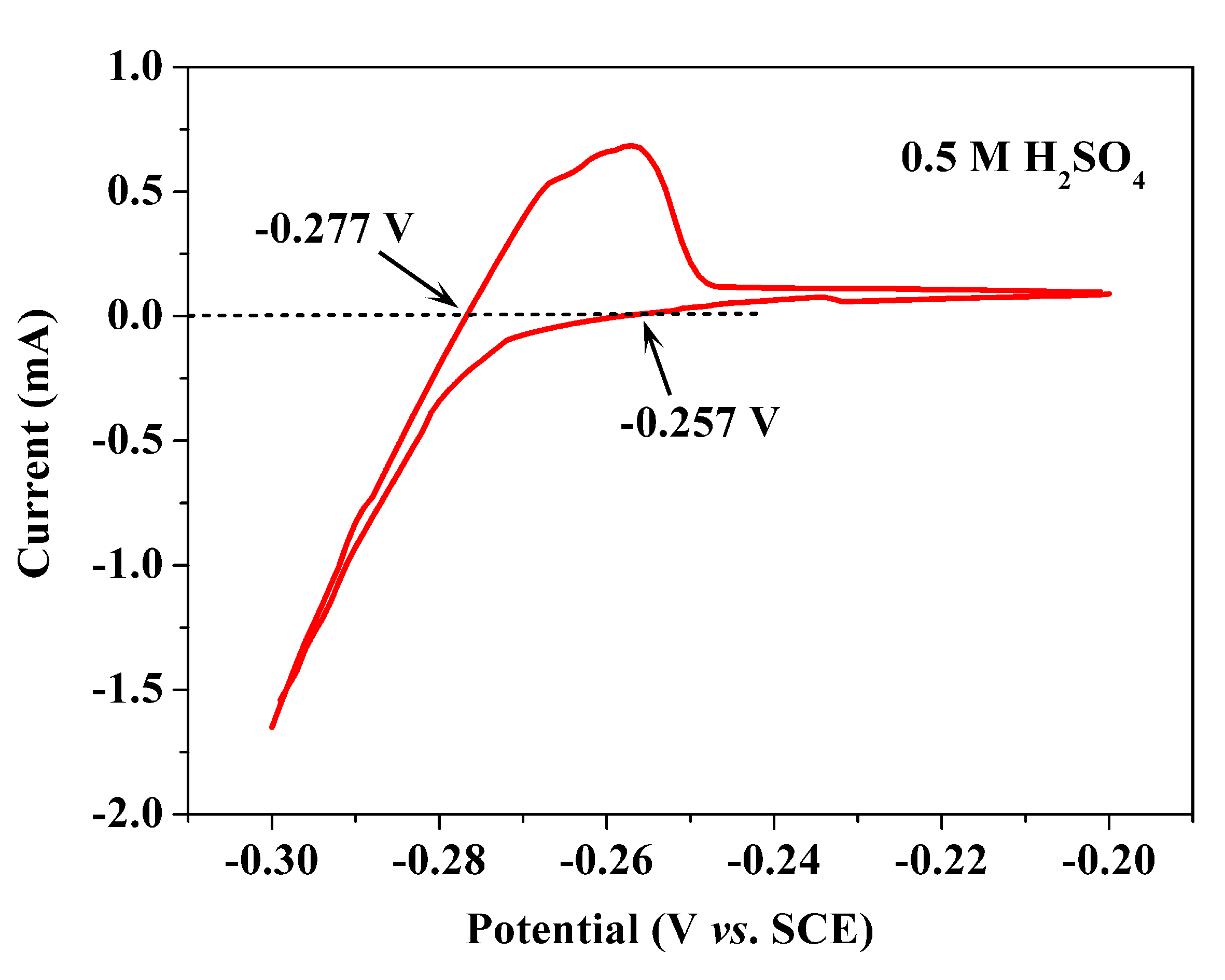


Figure S9. CV of Pt/C in hydrogen saturated 0.5 M H_2_SO_4_ solution, scan rate: 1 mV s^-1^.

Table S1. Total nitrogen content and percentage of different N species in Fe-N/C catalysts.

| **Sample** | **Total nitrogen content (at%)** | **Pyridinic N Pyrrolic N Graphite N Oxidized N**  **(%)** | | | |
| --- | --- | --- | --- | --- | --- |
| Fe-N/C NNs | 6.89 | 31.4 | 18.0 | 36.5 | 14.1 |
| Fe-N/C NMs | 6.86 | 38.7 | 24.1 | 24.9 | 12.3 |

Table S2. Comparison of ORR performance of different iron-based catalysts in acidic electrolyte.

It is well known that different electrochemical measurementmethods will affect the onset potential of electrocatalysts. Thus we chose ΔE_1/2_ as the judgment of eletrocatalytic performance. We defined ΔE_1/2_ = E_1/2_ (Pt/C) –E_1/2_ (Sample).

| **Sample** | **Catalyst loading**  **(mg cm^-2^)** | **ΔE_1/2_**  **(mV)** | **Current density at 0.4V *vs*. RHE**  **(mA cm^-2^)** | **Electrolyte** | **References** |
| --- | --- | --- | --- | --- | --- |
| Fe-N/C NNs | 0.32 | 0.108 | 3.9 | 0.5 M H_2_SO_4_ | This work |
| Fe-N-CNFs | 0.60 | 0.150 | 4.6 | 0.5 M H_2_SO_4_ | *Angew. Chem. Int. Ed.*,  2015, **54**, 1. |
| Pod(N)-FeCo | 3.10 | 0.301 | 3.1 | 0.1 M H_2_SO_4_ | *Angew. Chem. Int. Ed.*,  2012, **124**, 1. |
| 0.4-Fe-N-C | 0.42 | 0.090 | 5.4 | 0.1 M HClO_4_ | *Small*, 2014, **10**, 4072. |
| Fe-N/C-800 | 0.08 | 0.150 | 4.2 | 0.1 M HClO_4_ | *J. Am. Chem. Soc.*,  2015, **137**, 5555. |
| PANI-Fe/Silica colloid | 0.60 | 0.118 | 4.6 | 0.5 M H_2_SO_4_ | *J. Am. Chem. Soc.*,  2013, **135**, 16002. |
| Fe-N/C-800 | 0.10 | 0.059 | 5.6 | 0.1 M HClO_4_ | *J. Am. Chem. Soc.*,  2014, **136**, 11027. |
| Fe-PANI/C-Mela | 0.51 | 0.060 | 5.7 | 0.1 M HClO_4_ | *Sci. Rep.*,  2013, **3**, 1765. |
| N-Fe-MOF | 1.00 | 0.060 | 3.7 | 0.5 M H_2_SO_4_ | *Adv. Mater.*,  2014, **26**, 1378. |
